# Effects of submaximal and supramaximal accentuated eccentric loading on mass and function

**DOI:** 10.3389/fphys.2023.1176835

**Published:** 2023-06-28

**Authors:** Sergio Maroto-Izquierdo, Fernando Martín-Rivera, Kazunori Nosaka, Marco Beato, Javier González-Gallego, José A. de Paz

**Affiliations:** ^1^ i+HeATLH, European University Miguel de Cervantes (UEMC), Valladolid, Spain; ^2^ Research Group in Prevention and Health in Exercise and Sport, University of Valencia, Valencia, Spain; ^3^ Centre for Exercise and Sports Science Research, School of Medical and Health Sciences, Edith Cowan University, Joondalup, WA, Australia; ^4^ Institute of Health and Wellbeing, University of Suffolk, Ipswich, United Kingdom; ^5^ Institute of Biomedicine (IBIOMED), University of León, León, Spain

**Keywords:** eccentric-overload, strength, hypertrophy, muscle power, vertical jump, muscle endurance, anabolic hormones

## Abstract

**Introduction:** Eccentric-overload (EO) resistance training emerges as an alternative to more optimally prescribe intensity relative to the force generation capabilities of the eccentric muscle contraction. Given the difficulties to individually prescribe absolute eccentric loads relative to each person’s eccentric ability, setting the load relative to the concentric one-repetition maximum (1-RM) is the most used EO training approach. Therefore, we investigated the effects of submaximal and supramaximal (i.e., eccentric loads above 100% of 1-RM) accentuated eccentric training on changes in lean mass, anabolic hormonal responses and muscle function.

**Methods:** Physically active university students (*n* = 27) were randomly assigned to two training groups. Participants in the training groups performed dominant leg isotonic training twice a week for 10 weeks (four sets of eight repetitions). Isotonic resistance was generated by an electric-motor device at two different percentages of 1-RM for the eccentric phase; 90% submaximal load, SUB group) and 120% (supramaximal load, SUPRA group). Concentric load was the same for both groups (30% of 1-RM). Changes in total thigh lean mass (TTLM), anabolic hormonal responses (growth hormone, IGF-1, IL-6, and total testosterone), unilateral leg-press 1-RM, maximal voluntary isometric contractions (MVIC), local muscle endurance (XRM), muscle power at 40 (PP40), 60 (PP60) and 80% (PP80) of the 1-RM, and unilateral vertical jump height before and after training were compared between groups.

**Results:** After training, both SUB and SUPRA groups showed similar increases (*p* < 0.05) in MVIC (19.2% and 19.6%), XRM (53.8% and 23.8%), PP40 (16.2% and 15.7%), TTLM (2.5% and 4.2%), IGF-1 (10.0% and 14.1%) and IL-6 (58.6% and 28.6%). However, increases in 1-RM strength (16.3%) and unilateral vertical jump height (10.0%–13.4%) were observed for SUPRA only. Indeed, SUPRA was shown to be more favorable than SUB training for increasing 1-RM [ES = 0.77 (1.49–0.05)]. Unilateral muscle power at medium and high intensity (10.2% and 10.5%) also increased in SUB but without significant differences between groups.

**Discussion:** Similar functional and structural effects were demonstrated after 10 weeks EO training with submaximal and supramaximal eccentric loads. Although supramaximal loading might be superior for increasing 1-RM, the use of this approach does not appear to be necessary in healthy, active individuals.

## 1 Introduction

Resistance training (RT) is the most popular physical training intervention to enhance muscular strength, power and mass in both healthy and clinical populations (Kraemer et al., 2017), and the training intensity (i.e., mechanical load) used during training is known to be a key factor mediating adaptive responses (Schoenfeld et al., 2017). Traditional strength training is performed by the lifting (requiring concentric muscle actions) and lowering (requiring eccentric muscle actions) of weights, usually with the load being set at a specific proportion of an individual’s maximum strength capacity (Suchomel et al., 2018). However, muscles are not capable of lifting as much load in the concentric phase as they can lower with control in the eccentric phase due to the well-described force-velocity characteristics of muscles (Duchateau & Baudry, 2014; Duchateau & Enoka, 2016; Franchi et al., 2017). Therefore, the loads used during strength training are limited to those that can be lifted in the concentric phase, and the eccentric phase is never performed with maximal (or near-maximal) loads. Thus, a suboptimum stimulus is applied during the eccentric phase of strength training (Maroto-Izquierdo et al., 2022). Therefore, researchers, practitioners and strength and conditioning specialists have sought alternative methods in order to overload or accentuate the eccentric contraction.

Eccentric-overload (EO) RT emerges as an alternative to more optimally prescribe intensity relative to the force generation capabilities of the eccentric muscle contraction and avoiding the negative work isolation (i.e., favoring the strength-shortening cycle use) (Wagle et al., 2017). It consists of prescribing an eccentric load in excess of the concentric load (Schoenfeld & Grgic, 2018). Prior studies have reported evidence of force and power production enhancements (Aboodarda et al., 2013; Ojasto & Hakkinen, 2009; J. M; Sheppard & Young, 2010) and chronic adaptations using various systems to accentuate the eccentric loading, such as weight releasers (Walker et al., 2016; Walker et al., 2017; Walker et al., 2020), computer-driven devices (Friedmann et al., 2004; Yarrow et al., 2008; Friedmann-Bette et al., 2010; Maroto-Izquierdo et al., 2019; Magdi et al., 2021) or isoinertial flywheel devices (Maroto-Izquierdo et al., 2017a; de Keijzer et al., 2022). Recently, Walker et al. (2016) (Walker et al., 2017; Walker et al., 2020) demonstrated that EO RT with 40% greater load in the eccentric phase led to greater increases in maximum force production, muscle endurance capacity and muscle activation in comparison with traditional concentric-eccentric weight training, although anabolic hormonal responses (testosterone, cortisol, and growth hormone) and hypertrophic effects were similar to traditional strength training. English and collaborators (2014), showed increases in lean tissue mass (used as an estimate of muscle mass) only when EO (138% of the concentric load) was used during lengthening. In addition, Friedmann-Bette and co-workers (2010) found greater improvements in squat jump height and type IIa fiber cross-sectional area as well as a shift towards faster myosin heavy chain isoforms after 10 weeks of EO RT using an electric-motor device (1.9 times the concentric load during the eccentric action) when compared to traditional strength training. EO training with flywheel devices, in which eccentric loads exceed concentric loads by ∼25%, but with this overload being imposed only later in the eccentric phase (i.e., towards longer muscle lengths; which is a consequence of the system’s design) has also been shown to evoke greater adaptations on strength, power, hypertrophy and running speed than traditional strength training (Maroto-Izquierdo et al., 2017b; de Keijzer et al., 2022). Even though the eccentric load as a percentage of one-repetition maximum (1-RM) was the same for all the participants included in those studies, the absolute load relative to each person’s eccentric ability might be very different. Because of this, EO RT should be prescribed base on individual eccentric strength instead of maximum concentric ability. However, measuring eccentric strength accurately is very hard (especially with traditional training) since we can just move faster under heavier loads (Tomalka, 2023). Therefore, it is widely assumed that within the healthy, adult population there is some similarity in the eccentric-concentric strength ratio. Then, it is much easier to set the load relative to the concentric 1-RM. However, the ideal load on average to optimize the EO training-induced benefits remains undetermined.

Although throughout the scientific literature, loads ranging from 120% to 160% (Buskard et al., 2018) of concentric 1-RM have been prescribed for performing accentuated eccentric exercise, some authors have recommended that the ideal load exercise is 120% of the concentric 1-RM (Schoenfeld & Grgic, 2018). Indeed, supramaximal loading (i.e., loads above 100% of the concentric 1-RM) is the most commonly used strategy. However, several studies have found similar adaptations with loads lower than 100% of the 1-RM (i.e., submaximal loads) (Friedmann et al., 2004; English et al., 2014; Krentz et al., 2017; Buskard et al., 2018). Thus, although supramaximal EO training has been shown to be effective at increasing muscle strength, it did not appear to be more effective than traditional training at increasing lower body 1-RM (Buskard et al., 2018). Moreover, concerns regarding injury risk, muscle damage, and null benefit over traditional methods may limit the practical utility of this approach (Buskard et al., 2018). Hence, we hypothesize that it is not necessary to employ eccentric loads greater than the concentric 1-RM during EO RT to achieve significant adaptations. However, as far as our knowledge is concerned, the optimal amount of eccentric loading during EO training remains unknown, as well as the comparison between supramaximal and submaximal loading training-induced effects. Therefore, this study aimed to compare the effects of supramaximal and submaximal accentuated eccentric loading on lean tissue mass, neuromuscular performance and anabolic hormones responses in physical active men.

## 2 Material and methods

### 2.1 General design

Male sports science undergraduate students (*n* = 27) volunteered for the study and were randomly allocated to one of the two training groups. Participants completed 20 sessions (four sets x eight repetitions) of the unilateral leg press exercise with EO over 10 weeks for the dominant leg. Isotonic resistance was generated by an electric-motor device at two different percentages of the concentric 1-RM for the eccentric phase; 90% (submaximal load, SUB group) or 120% (supramaximal load, SUPRA group). Concentric load was the same for both groups (30% of 1-RM). Unilateral leg press 1-RM and maximal voluntary isometric contraction (MVIC), muscle local endurance, unilateral muscle power at different percentages of the 1-RM, unilateral vertical jump, thigh lean tissue mass (TTLM) and anabolic hormones responses were assessed before and after training intervention. Participants came to the lab in four occasions prior to start the training programme. On day 1, dual energy X-ray absorptiometry, venus blood samples and vertical jump tests were completed. 24 h later MVIC and 1-RM tests were performed. 48 h later, mechanical power and muscle endurance tests were conducted. One week after training, these protocols were replicated in the same order and at the same time. Each day that participants attended to the laboratory, a warm-up of 5-min cycling, 25 reps of high knee, 25 reps of butt kicks and 2 sets of 10 squat repetitions with their own body weight was performed.

### 2.2 Participants

Thirty sports science undergraduate students volunteered for the study (20.1 ± 2.1 years, 75.6 ± 7.9 kg and 178.8 ± 5.3 cm). Participants were moderately active and healthy, and engaged in 6–8 h of recreational physical activities per week. They had history of regular lower limb strength training for at least 1 year and no muscle joint or bone injury in the last 6 months. They were informed of the purposes and risks involved in the study before giving their informed written consent to participate. They were asked not to change their exercise habits and not to perform resistance exercises during the experimental phase of the study. The Ethics Committee of the University of León approved the study protocols (ETICA-ULE-009-2018a). Three participants dropped out of the study: one due to an accident and two due to scheduling conflicts. The remaining participants (*n* = 27) completed all study procedures as planned, including pre and post-testing sessions, familiarization sessions, and the 20 training sessions.

### 2.3 Training program

**FIGURE 1:**
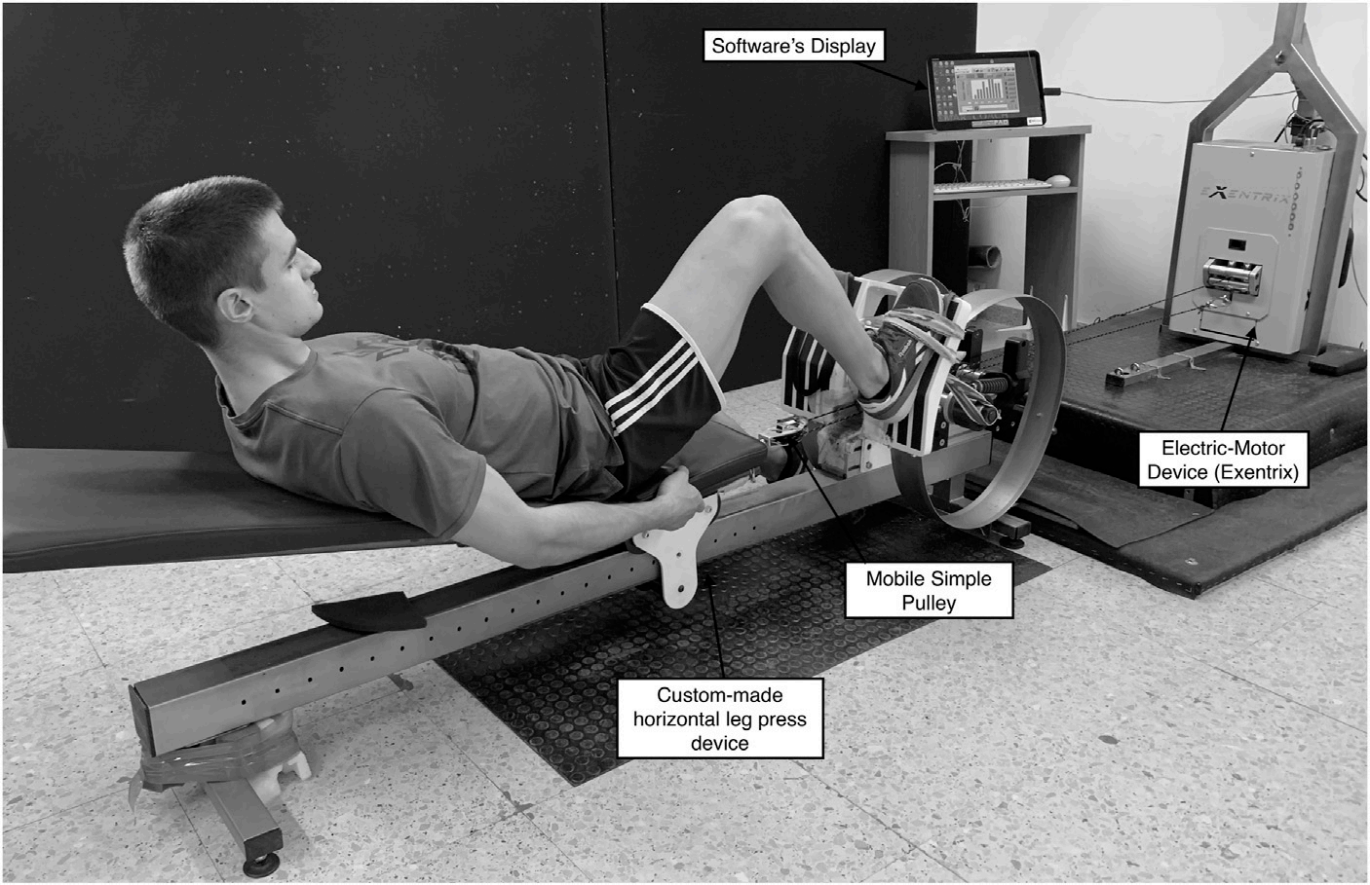
Depicts the setup of the leg press exercise on the electric-motor device.

All participants [SUB (*n* = 14); and SUPRA (*n* = 13) groups] completed 10-weeks (20 sessions) of an eccentrically accentuated unilateral leg press training program, using an electric-motor device (Exentrix, SmartCoach™, Stockholm, Sweden) (Figure 1). Volunteers trained twice a week with at least 48 h of rest between sessions. Following a standardized cycling warm-up, participants performed four sets of eight maximal unilateral (dominant leg) coupled concentric and eccentric muscle actions in a custom-made horizontal leg press device. The electric-motor training device was configured in isotonic mode (i.e., constant load during exercise) using the device’s software settings (Exentrix PC Interface—V2.4, SmartCoach™). Hence, two different intensities were employed for each group: a submaximal load of 90% of the 1-RM (SUB group) (Ojasto & Hakkinen, 2009) and a supramaximal load of 120% of the 1-RM (SUPRA group) (Schoenfeld & Grgic, 2018), while no different intensities were selected for the concentric phase for any group (30% of the 1-RM) (Friedmann et al., 2004; English et al., 2014). According to the manufacturer’s instructions, a single hoist with a mobile simple pulley was employed to duplicate the force generated by the electric-motor and apply the prescribed intensity for each participant (see Figure 1). In addition, the transition time between eccentric and concentric action was the minimum allowed by the system (0.5 s). Participants were required to push with maximal effort through the entire range of motion (ROM), which ranged from 90°-knee flexion to nearly full extension (0°-knee flexion). At the end of the concentric contraction, the motor strap rewound back, initiating the reversed braking action. Before each session, the ROM for each participant was set up from 0° to 90°-knee flexion using a goniometer. Then, the only instruction given to participants was to stop the movement before reaching the end of the ROM. Participants were blinded regarding the load condition and they were not allowed to use the other leg to produce force, They were instructed to keep the non-exercised leg fully extended and supported on a slider, allowing the foot of the non-trained leg to slide along the floor without exerting any resistance during the execution of the trained leg (Figure 1).

The electric-motor providing the resistance (a brushless DC motor) was controlled by a custom-designed power driver (SmartCoach™, Stockholm, Sweden), that controls in closed loop both speed and torque variables, allowing for a precise load prescription for each contraction. For the speed control loop, a high precision incremental encoder is used to measure the actual speed. For the torque control loop, the current measured in the motor winding is used instead. With a technique used in automation and robotics, the actual torque (T, Nm) is computed from the electric current. The motor is coupled directly to a steel shaft (D = 25 mm) over which the rope is wound. Since the shaft is supported by a low-friction ball bearing, and the coupling between subject and motor is direct (no gears, no pulleys), the force can be computed by dividing the torque by the lever arm b = D/2 + d/2, where d is the rope diameter. Hence, F = T/b. Thus, mean and peak force and power were measured during each concentric an eccentric contraction, and real-time feedback was provided on a computer monitor. A strong verbal encouragement was given during each repetition performed, as well as power was measured by an integrated encoder during each repetition (concentric and eccentric muscle actions; Exentrix PC Interface-V2.4, SmartCoachTM), and real-time feedback was provided on a computer monitor to ensure that each repetition was performed with the maximum movement intention and that the development of concentric peak power remained stable during each set and between sets in each training session. All participants were familiarized over two sessions (the first familiarization session consisted of four sets of eight reps with an intensity of 30% of the 1-RM in both concentric and eccentric actions to learn the technique and familiarize with the training device, and the second familiarization session consisted of three sets of eight reps with an intensity of 30% during the concentric contraction and 80%–120% of the 1-RM during the eccentric contraction) with the single-leg squat exercise ∼1 week before the first training session.

### 2.4 Procedures

#### 2.4.1 Lean tissue mass

Dual energy X-ray absorptiometry (DXA) was performed ∼1 week before the first training session and 1 week after the last training session, at the same time of the day, using a Lunar Prodigy^®^ whole-body scan (GE Medical Systems, Madison, WI) accordingly with the protocols described by Nana et al. (2016); Tavoian et al. (2019), i.e., participants were encouraged to have a similar sleeping time and eating for both scans and to avoid any exercise on the morning of the scan. They were advised to report to the laboratory in a euhydrated state, fasted overnight and with the bladder voided. They were asked to wear underwear and to remove all jewelry and metal objects. Care was taken to follow The International Society for Clinical Densitometry guidelines for positioning during the scan (Hangartner et al., 2013). A manual analysis was performed to estimate total thigh lean mass of each leg following a previously described protocol (Maroto-Izquierdo et al., 2019). Briefly, one rectangle mark was generated using the lower margin of the ischial tuberosities and the lower margin of the femoral condyles as thigh reference points. Lean mass was then calculated for the entire thigh. Subsequently, inside the span of the thigh rectangle a perpendicular line of half minus 1 cm of the length of the rectangle was drawn from the distal to the proximal mark to establish a region of interest (ROI) of the thigh where lean muscle mass was estimated. Then, a 20 mm-thick slice was placed above this vertical line (i.e., medial thigh ROI) (Fernandez-Gonzalo et al., 2014). In addition, two other vertical lines of half minus 1 cm of the length between the proximal and distal margins of the medial ROI rectangle and the proximal and distal horizontal marks of the initial rectangle were drawn from proximal and distal medial thigh ROI’s lines respectively. Thus, two 20-mm slices were placed above (i.e., proximal thigh ROI) and below (i.e., distal thigh ROI) the first slice. Finally, lean tissue mass estimation in both total thigh and the three slices created was calculated using Encore software for both training and non-training legs. Lean tissue mass estimation in each thigh was calculated using the device’s software (Encore^®^ 2009 software, Lunar Corp., Madison, WI). The error attributable to manual positioning of the region of interest limits, assessed from repeated analysis of the scans of twenty random subjects, was 1.0%.

#### 2.4.2 Hormonal responses

Venus blood samples (30 mL) were obtained immediately after DXA scan, in the early morning in the fasted state, from the cephalic vein using Vacutainer^®^ plus plastic serum tubes with spray-coated silica (BD, Franklin Lakes, NJ, United States) 7 days before and after the training intervention in a subset of 20 participants (10 patients randomly assigned from each group). To avoid circadian effects, all samples were collected between 08:00 and 10:00 a.m. Participants were required to avoid any intense exercise during the previous 5–6 days. No caffeine or alcohol was allowed 48 h prior to blood extraction. Peripheral blood was rested for 1 h to allow its coagulation. Serum was isolated by centrifugation of 1 h-clotted peripheral blood at 1,200 rpm during 10 min at 4°C, and then was stored at −80°C until further use. Growth hormone (GH), and insulin growth factor 1(IGF-1) and interleukin 6 (IL-6) and total testosterone, were analyzed using chemiluminescence techniques (Immulite 1,000, Siemens, Illinois, United States). Assay’s sensitivities for these hormones were: T = 0.5 nmol.L1, 22 GH = 0.01 mgL1, C = 5.5 nmol·L^−1^. Each subject’s samples were analyzed in the same run.

#### 2.4.3 Unilateral vertical jump performance

Vertical jump tests were completed after the DXA scan and blood draws. After warm-up, jump height was measured for three jump types performed unilaterally on a contact platform (Globus Ergotester^®^, Globus, Codogne, Italy): squat jump (SJ), countermovement jump (CMJ), and drop jump (DJ). SJ was performed from a 90° knee flexion with hands on the hips. For CMJ, participants started in a standing straight position and were instructed to jump as high as possible with hands on the hips. In DJ, participants stepped off the platform of a box of 45 cm above the ground and then jump as high as possible immediately after landing. Jump height and DJ contact time were measured with a precision of 0.1 cm (0.05 s for contact time) and demonstrated a high level of partial reliability (ICC = 0.98, 95% CI: 0.95–0.99) across all the tests administered. Three trials, with 30 s recovery, were allowed and the best result was included in the data analysis.

#### 2.4.4 Unilateral maximal voluntary isometric force

Twenty-four hours later, the unilateral maximal voluntary isometric contraction (MVIC) strength was assessed at 90°-knee flexion on a 45° inclined leg press device (Gerva-Sport, Madrid, Spain). The leg press device was equipped with a force transducer (Omega M285291, S-Beam KM 1,506 K, Art No. 124.108, Megatron Elektronic AG, Putzbrunn, D, input voltage: ± 5 V). The sensor was integrated in the security chain between the weights bar and the leg press seat, parallel to the 45°-inclined middle rail. Participants were instructed to perform two 5-s ramped knee extension isometric contractions. Verbal instructions to perform each contraction with maximum effort were given continuously. Data from the force transducer were sampled at 1,000 Hz and a 2-min recovery period between attempts was allowed. Only the best repetition performed was used for further analysis.

#### 2.4.5 Unilateral maximal dynamic force

Immediately after MVIC test, the unilateral one-repetition maximum (1-RM) load in the leg press exercise was assessed on the same 45° leg press device described above. Each participant performed the 1-RM test from full knee extension (0°) to 90° flexion and then extending to full extension with a load corresponding to approximately 3-RM. The load was increased by 10 kg if the participant succeeded or decreased 5 kg if they failed. Testing ended when each participant was unable to overcome a given load in two successive trials. Unilateral 1-RM load was achieved between 3 and 6 attempts, and trials were interspersed by a 2-min recovery. Participants were asked to place the untested leg with the knee flexed and the foot propped on the ground. The previously described warm-up was implemented with one set at ∼8RM load. The partial reliability (intraclass correlation coefficient, ICC) was 0.98 (95% CI: 0.93–0.99).

#### 2.4.6 Unilateral maximal dynamic power

Forty-eight hours after the 1-RM test, each participant completed three sets of three unilateral repetitions from full knee extension (lowered with control) to 90° flexion and then extending to full extension (0°) in the leg press, as described above, with a 2-min recovery between sets. To avoid use of the stretch-shortening cycle, each repetition started from a static position (load was individually locked at the exact point of 90° knee flexion by a security strap). The warm-up protocol described for the 1-RM test was also performed before the muscle power test. Sets were performed at 40, 60, and 80% of 1-RM, and the order of the sets were individually randomized before testing and replicated at post-training testing. Participants were asked to perform the concentric phase of each repetition as fast as possible. Concentric peak power data for each repetition were captured at 1,000 Hz using an encoder (T-FORCE Dynamic Measurement System, Ergotech Consulting S.L., Murcia, Spain) and the associated software (T-Force v. 2.28). The best repetition performed at each load was used for data analysis. Partial reliability (ICC) for unilateral concentric peak power was high across all loads [40%: 0.91 (95% CI: 0.81–0.96); 60%: 0.93 (95% CI: 0.86–0.97); 80%: 0.90 (95% CI: 0.80–0.96)].

#### 2.4.7 Unilateral leg press muscle endurance

Once the mechanical power tests were done, participants were asked to perform the 45°-inclined leg press repetition-to-failure test with 60% of their 1-RM load using the dominant leg. Thus, the same relative load, but different absolute load, was lifted before vs. after the training period. Subjects were instructed to maintain a cadence of 2 s concentric and 2 s eccentric phases, which was monitored by the investigator, and to perform as many repetitions as possible. All repetitions completed between 90°-knee flexion and full extension (i.e., 0°-knee flexion) were considered. The test was terminated when the subject was unable to lift the load until total knee extension for two successive repetitions and only successful repetitions were used in further analysis. The encoder described above (T-FORCE Dynamic Measurement System, Ergotech Consulting S.L., Murcia, Spain) and the associated software (T-Force v. 2.28) were used to quantify the number of repetitions completed and the distance in each repetition. Minimal recovery periods between repetitions were not allowed. The technical execution was controlled by an individual researcher and subjects were strongly encouraged. A single maximum attempt was completed, and the absolute number of repetitions performed was recorded.

#### 2.4.8 Muscle soreness

Additionally, immediately after, and 2, 24, and 48 h after training sessions 1, 10, and 20 perceived muscle soreness was assessed using a 0–10 visual analog scale (VAS) with a 100-mm horizontal line with “no pain” on one end (0 mm, 0 points) and “extremely painful” on the other (100 mm, 10 points). Participants were asked to mark the perceived pain level during a functional activity (i.e., standing to sitting position) on the VAS.

### 2.5 Statistical analyses

All statistical analysis was performed using the Jamovi software package (The Jamovi Project, v.1.6.23.0; downloadable at https://www.jamovi.org). Normality was checked by the Shapiro-Wilk normality test. Then, a repeated measures linear-mixed model fitted with a restricted maximum likelihood method and unstructured covariates with a Tukey *post hoc* adjustment was used to compare outcomes between time (pre and post) and training protocol (SUB and SUPRA). The effect size (ES) was calculated for interactions between groups using Cohen’s guidelines. Threshold values for ES were >0.2 (small), >0.6 (large), and >2.0 (very large) (Hopkins et al., 2009). Mean, standard error (SE) and the t value were reported for all statistical analyses. Additionally, for the between-group analysis, a customized spreadsheet (Hopkins, 2007) was employed to convert the ANCOVA *p*-values and the effect statistic to magnitude-based inferences. To make inferences about the true values of the effect on the variables assessed, 95% confidence intervals (CI) were used. An effect was considered unclear if its CI simultaneously overlapped the thresholds for positive and negative or if the chances of the effect being substantial for SUB group or for SUPRA group were both >5%. The qualitative terms and the default values were: most unlikely, <0.5%; very unlikely, 0.5%–4.9%; unlikely, 5.0%–24.9%; possibly, 25.0%–74.9%; likely, 75.0%–94.9%; very likely, 95.0%–99.5%; and most likely, >99.5% (Hopkins, 2007). The significance level was set to *p* < 0.05.

## 3 Results

The average peak power for both concentric and eccentric phase increased (*p* < 0.01) from the first to the last training session, but the increases were similar between SUB (concentric: 475.6 ± 68.6–655.1 ± 81.3 W, 38%; eccentric: 842.1 ± 81.5–1,053.1 ± 70.7 W, 25%) and SUPRA (concentric: 490.5 ± 85.7–666.5 ± 73.6 W, 36%; eccentric: 888.2 ± 122.1–1,095.2 ± 61.0 W, 23%). The mean concentric velocity (2.89 ± 0.30 m/s) were similar between both SUB and SUPRA training groups, but the mean eccentric phase velocity was faster (*p* < 0.05) in SUPRA (1.16 ± 0.22–1.61 ± 0.31 m/s) than SUB (1.11 ± 0.26–1.34 ± 0.19 m/s). However, no significant differences were found in the average duration of each contraction between SUB (concentric: 0.29 ± 0.04 s, eccentric: 1.39 ± 0.28 s) and SUPRA (concentric: 0.28 ± 0.04 s, eccentric: 1.32 ± 0.30 s).

Both training groups (SUB and SUPRA) increased total thigh lean mass (*p* < 0.01) after the 10-week training period (Table 1). The thigh lean mass increased by 2.5%–6.2% (*p* < 0.05) for all testing measures in both SUB (95% CI = −19.9–377.1, *t* = 4.33–5.69, ES: 0.35–0.54) and SUPRA groups (95% CI = −24.1–367.3, *t* = 2.90–5.16, ES: 0.31–0.60). In addition, when comparing the lean tissue mass changes between groups, significant differences (*p* < 0.05) were observed at post-training between TTLM (*t* = 3.00) and thigh lean mass at distal level (*t* = 2.94), although statistical differences (*p* < 0.05, *t* = 3.19) were also observed before intervention on TTLM.

**TABLE 1:** Changes (mean ± SD) in unilateral total thigh lean mass (TTLM), for the trained legs for the both experimental group [Submaximal and supramaximal groups)] before (Pre) and after training (Post), *p*-value for the comparison between pre- and post-training values by Tukey test, and effect size (ES) and magnitude of change (%) and 95% CI.

	Pre	Post	*p*		ES	%	95% CI for difference		
							Mean dif	Lower	Upper
Supramaximal group	(*n* = 14)								
TTLM (g)	6,821.0 ± 441.5^b^	7,096.2 ± 443.4^b^ ^,^ ^c^	<0.001	***	0.39	2.5	275.2	173.4	377.1
TLM -P (g)	462.3 ± 59.0	482.1 ± 54.2	0.002	**	0.35	4.3	19.9	10.4	29.3
TLM -M (g)	393.7 ± 36.5	409.8 ± 44.7	0.001	**	0.39	4.1	16.1	−19.9	52.1
TLM -D (g)	227.0 ± 19.6	237.5 ± 19.3^b^ ^,^ ^c^	<0.001	***	0.54	4.6	10.5	5.9	15.1
Supramaximal group	(*n* = 13)								
TTLM (g)	6,214.4 ± 543.2	6,476.0 ± 622.0	<0.001	***	0.45	4.2	261.6	156.0	367.3
TLM-P (g)	444.3 ± 36.6	459.1 ± 44.4	0.030	^a^	0.36	3.3	14.8	5.0	24.6
TLM -M (g)	357.5 ± 36.0	379.7 ± 37.5	0.011	^a^	0.60	6.2	13.2	−24.2	50.5
TLM -D (g)	207.1 ± 23.3	214.1 ± 22.1	0.016	^a^	0.31	3.4	7.0	2.2	11.8

Abbreviations: TLM, thigh lean mass; TTLM, total thigh lean mass; D, distal; M, medial; P, proximal.

^a^ Significant (*p* < 0.05) difference from the pre-training value.

^b^ Significant (*p* < 0.05) difference from the other group at pre-training.

^c^ Significant (*p* < 0.05) difference from the other group at post-training.

**FIGURE 2:**
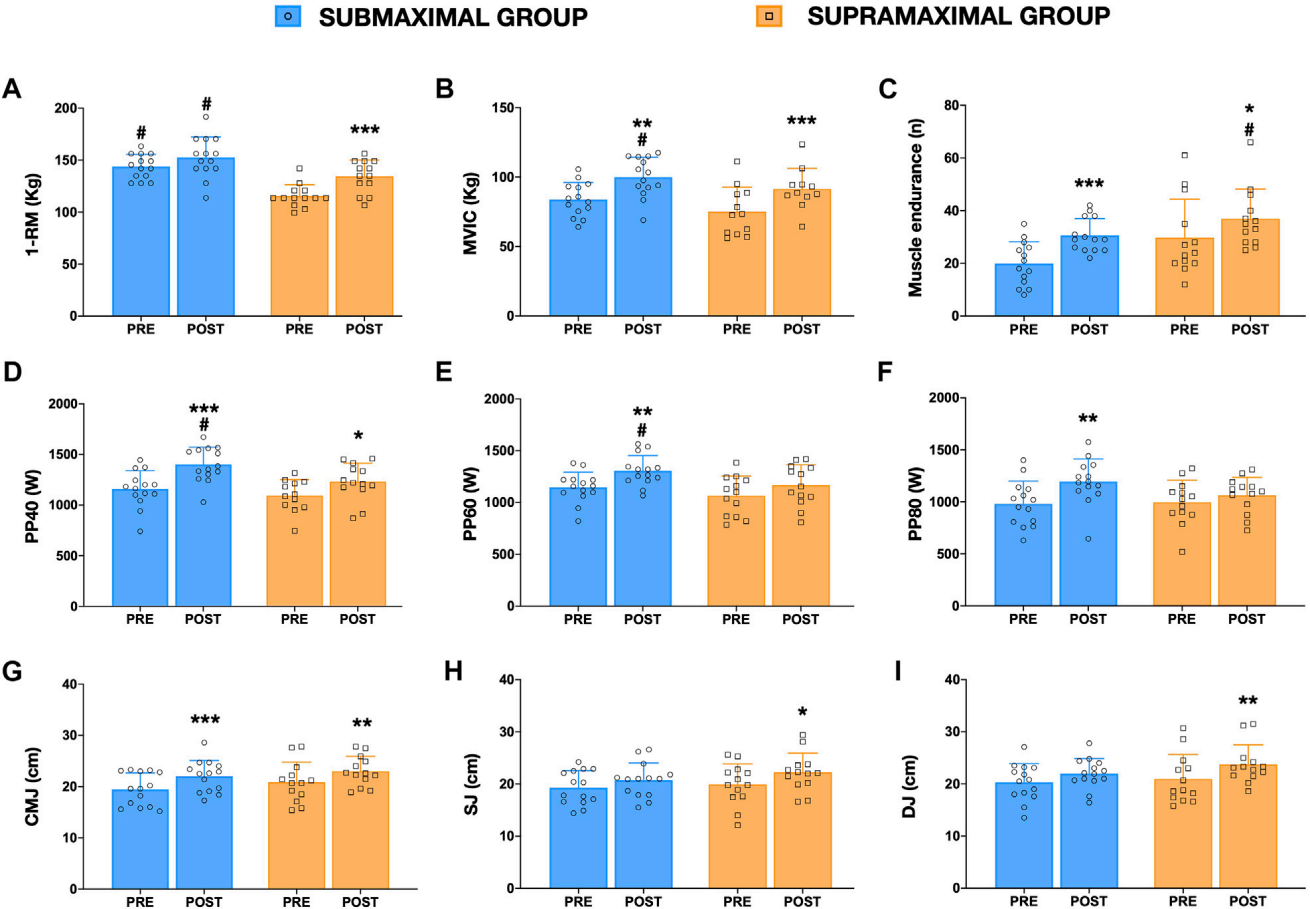
Mean ± SD values in one-repetition maximal of one leg press [1-RM, **(A)]**, maximal voluntary isometric contraction [MVIC, **(B)]**, local muscle endurance [XRM, **(C)]**, concentric peack power at 40% [PP40, **(D)]**, 60% [P6P0, **(E)]** and 80% of 1-RM [PP80, **(F)]**, countermovement jump height [CMJ, **(G)]**, squat jump height [SJ, **(H)]** and drop jump height [DJ, **(I)]** for SUB (blue) and SUPRA (orange) i groups. *A significant (*p* < 0.05) change from the baseline. ^#^A significant (*p* < 0.05) difference from the other group at pre-training.

**FIGURE 3:**
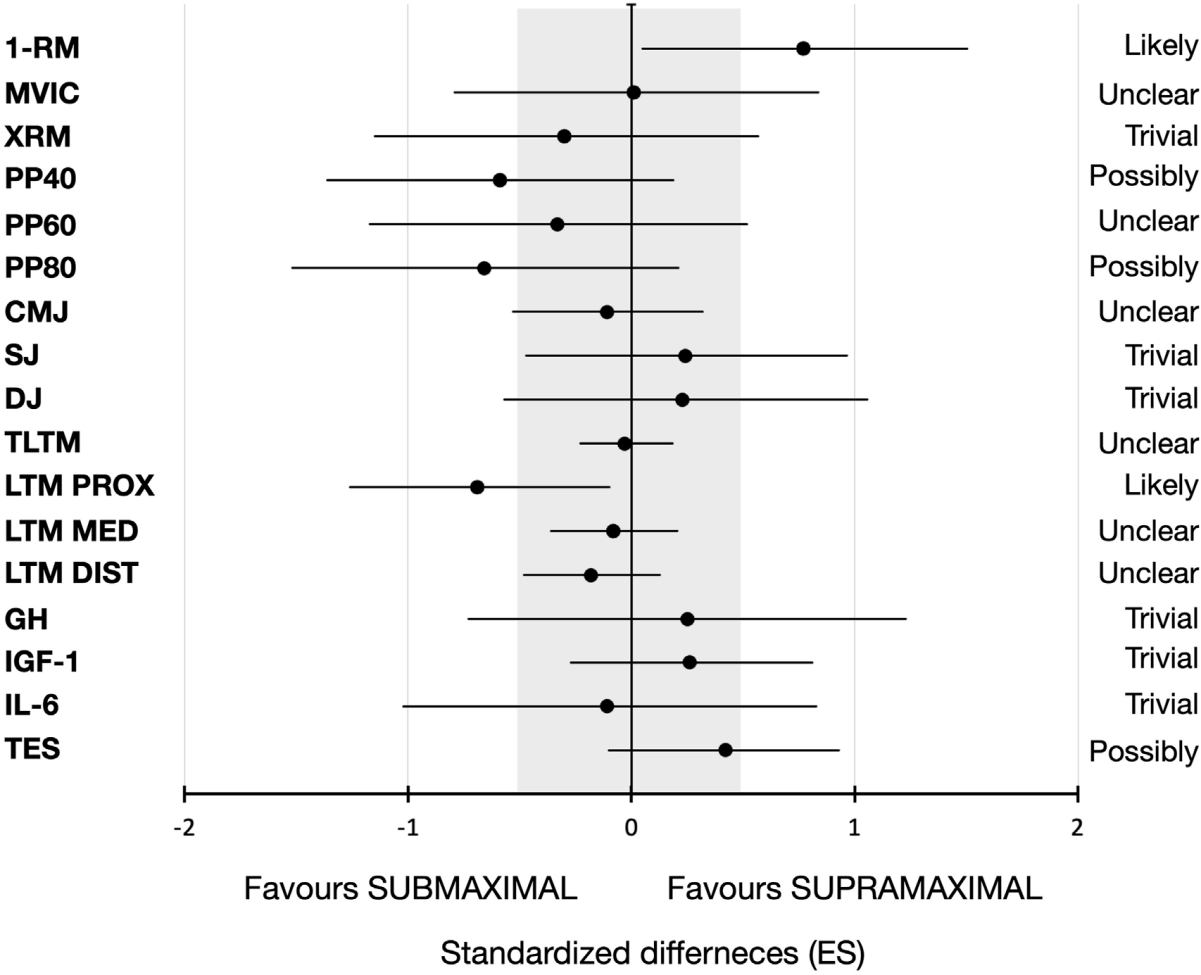
Standardized differences and its upper and lower limits to represent the efficiency of the supramaximal loading strategy compared to submaximal loading on changes in total thigh lean mass (TTLM) and at proximal (PROX), medial (MED), and distal (DIST) thigh levels, anabolic hormonal responses [growth hormone (GH), insulin growth factor 1 (IGF-1), interleukin-6 (IL-6), and total testosterone (TES)], unilateral leg-press maximum strength (1-RM), maximal voluntary isometric contractions (MVIC), local muscle endurance (XRM), muscle power at 40% (PP40), 60% (PP60), and 80% (PP80) of the 1-RM, and unilateral vertical jump (CMJ, SJ, and DJ) height before and after training.

Functional changes are shown in Figure 2. Regarding muscular 1-RM (Figure 2A), only SUPRA demonstrated significant increases after training (16.3%, *p* < 0.001, SE = 3.7, *t* = 5.0, ES = 1.39). In addition, significant differences (*p* < 0.05) were observed between groups when pre and post-training SUB values were compared with pre-training SUPRA 1-RM. Although no significant differences were observed after training between groups, standardized ES differences analysis showed that supramaximal loading strategy seems to be more favorable for increasing the 1-RM [ES = 0.77 (1.49–0.05), Figure 3]. However, both groups showed significant increases in MVIC (Figure 2B; SUB: 19.2%, *p* = 0.003, SE = 3.98, *t* = 4.1, ES = 1.21; SUPRA: 27.3%, *p* = 0.001, SE = 4.50, *t* = 4.4, ES = 1.36) and local muscle endurance (Figure 2C; SUB: 53.8%, *p* = 0.001, SE = 2.51, *t* = 4.3, ES = 1.44; SUPRA: 23.8%, *p* = 0.049, SE = 2.61, *t* = 2.7, ES = 0.54). Indeed, higher (*p* < 0.001, *t* = 5.1) post-training MVIC values were observed for SUB compared to SUPRA pre-training values, and greater (*p* < 0.001, *t* = 4.3) local muscle endurance values were observed after training in the SUPRA group when compared to pre-training SUB values. As shown in Figure 2D, both training groups showed significant (*p* < 0.05) increases in concentric peak power at 40% of 1-RM (SUB: 21.0%, SE: 40.1, *t* = 6.1, ES = 1.44; SUPRA: 12.6%, SE = 43.3, *t* = 3.3, ES = 1.16). However, at PP60 (14.0%, *p* = 0.007, SE = 58.9, *t* = 3.6, ES = 0.97) and PP80 (21.7%, *p* = 0.003, SE = 40.1, *t* = 4.0, ES = 1.17) significant increases were observed for SUB only (see Figures 2E, F). Significant differences between groups (*p* < 0.05) were only observed between SUB post-training and SUPRA pre-training values in PP40 and PP60. However, regarding vertical jump (Figures 2G–I), only SUPRA showed significant vertical jump height increases after training in all tests (CMJ: 10.0%, *p* = 0.008, SE = 0.60, *t* = 3.5, ES = 0.61; SJ: 11.6%, *p* = 0.011, SE = 0.69, *t* = 3.4, ES = 0.61; and DJ: 13.4%, *p* = 0.010, SE = 0.80, *t* = 3.5, ES = 0.66). But SUB only improved CMJ vertical jump height (13.4%, *p* < 0.001, SE = 0.58, *t* = 4.4, ES = 0.83). No significant differences on vertical jump height were observed between groups at any time.

**FIGURE 4:**
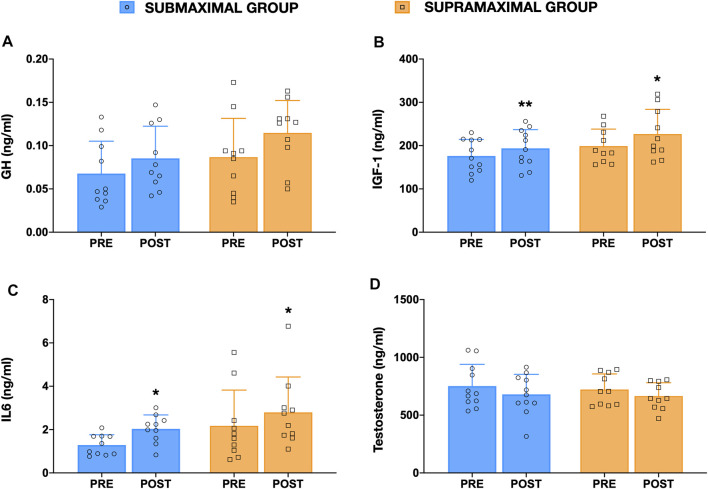
Growth hormone (GH), interleukin-6 (IL-6), insulin growth factor 1 (IGF-1) and total testosterone concentration before (pre) and after (post) training for both SUB and SUPRA experimental groups. * Significantly different (*p* < 0.05) from pre-training value.

Hormonal responses are displayed in Figure 4. Post Hoc analysis showed a significant time interaction for GH (*p* < 0.05, F = 5.7), IGF-1 (*p* < 0.01, F = 13.3), IL-6 (*p* < 0.001, F = 17.8) and total testosterone (*p* < 0.05, F = 5.6) concentration. In addition, significant time*group interactions were observed for IGF-1 and IL-6 (Figure 4B) in both SUB (IGF-1: *p* = 0.002, 17.6, SE = 8.6, ES = 1.22, *t* = 3.82; IL-6: *p* = 0.008, 0.75, SE = 0.22, ES = 1.07, *t* = 3.13) and SUPRA (IFG-1: *p* = 0.029, 27.7, SE = 8.90, ES = 0.72, *t* = 2.48; IL-6: *p* = 0.029, 0.62, SE = 0.24, ES = 0.82, *t* = 2.60) groups experienced a significant increased after training. But no statistical differences between groups were detected for other variables. There were no between-group significant differences for any hormonal response measured at pre- and post-training levels. No significant correlations were observed when changes in any hormone concentration was compared to strength-related and lean muscle mass-related gains. Finally, regarding muscle soreness, no significant differences were observed between groups at any time for any training session (please see Supplementary File S1).

## 4 Discussion

The aim of this study was to compare the effects of submaximal and supramaximal loading during EO RT on lower-limb neuromuscular performance, lean tissue mass and anabolic hormonal responses in healthy, physical active men. After 10 weeks (20 sessions), EO RT led to comparable increases in MVIC, muscle local endurance, unilateral muscle power at low intensity, thigh lean mass, IGF-1 and IL-6 in both SUB and SUPRA groups. However, increases in 1-RM strength and unilateral vertical jump height (SJ and DJ) were observed for SUPRA only. Indeed, SUPRA was shown to be more favorable than SUB training for increasing 1-RM (ES = 0.77, 95% CI = 1.49–0.05). Unilateral muscle power at medium and high intensity (10.2% and 10.5%) also increased only in SUB but without significant differences between training groups. No significant differences were observed between groups after training. Nonetheless, when considering a supramaximal load of 120%, it appears that accentuated eccentric loading with loads above the concentric 1-RM does not have a major impact on training-induced changes in comparison with submaximal loading.

Accentuated eccentric loading has been proposed as an alternative method to optimize RT due to lesser recruitment and discharge rates observed during eccentric contractions when compared to concentric contractions under similar absolute loading, which provides justification for higher absolute eccentric loading (Franchi et al., 2017). Furthermore, EO RT has demonstrated to increase force production in the subsequent concentric action, not only by motor cortex activation and spinal inhibition occurred during eccentric contractions, but also by a selective recruitment of high threshold motor units (Duchateau & Enoka, 2016) leading to better neuromuscular function. In addition, architectural muscle gearing may also contribute to these greater force production mechanisms, since muscle fascicle functions closer to its optimal length and angle (Ando et al., 2018). By means of this approach, stimulation of Type Ia afferent nerves will occur, inducing a myostatic reflex, and elastic energy stored in series and parallel elastic components during lengthening will be used in the subsequent concentric contraction, which is also due to the titin protein contribution (Hessel et al., 2017). Thus, after accentuated eccentric loading training neuromuscular adaptations may be attained that favor greater increases in strength and power compared to traditional training (Kaminski et al., 1998; Brandenburg & Docherty, 2002; Barstow et al., 2003; Friedmann et al., 2004; Yarrow et al., 2008; Friedmann-Bette et al., 2010; English et al., 2014; Walker et al., 2016; 2017; 2020; Douglas et al., 2018; Maroto-Izquierdo et al., 2019; Magdi et al., 2021).

In this study, only supramaximal loading led to significant increases in dynamic maximum muscle strength, with increases of 6% (SUB) and 16% (SUPRA) in 1-RM. Despite the SUB group showed higher strength levels at pre-training, these differences between groups were attenuated after training due to the greater improvements experienced by SUPRA. These results showed a greater training effect of supramaximal eccentric loads during EO-RT in comparison with submaximal loads. In addition, magnitude-based inference analysis showed likely large effects in 1-RM after training with supramaximal EO, whereas submaximal loading led to likely small effects. Likewise, a prior study with a similar submaximal loading design (isotonic concentric load of 30% of the 1-RM, and isotonic eccentric load of 70% of the 1-RM), showed comparable muscle torque enhancements (5%) after training (Friedmann et al., 2004). Although no differences between the EO-RT group and the traditional RT group (30/30% 1-RM) were observed, the traditional group did not show significant improvements in muscular strength (Friedmann et al., 2004). Therefore, these gains might be attributed to the EO applied during RT (Wagle et al., 2017). Regarding supramaximal loading training, Yarrow and co-workers (2008), showed similar results in lower limb 1-RM (gains of 19%) after training with supramaximal loads (upon 120% of the 1-RM) with high EO percentages (concentric load of ∼45% of the 1-RM) employing free-weights and an electric-motor device to raise the weight during the concentric phase. Similarly, Friedmann-Bette et al. (2010) demonstrated significant increases of 11–15 kg in the 1-RM test when a supramaximal loading strategy was applied with an electric-motor device in the knee extensor muscles. Interestingly, in the study mentioned above, the concentric load was higher (8RM) in comparison with our experimental design, resulting in similar gains, or even lower gains with a higher total volume load. These results suggest that EO-RT with higher EO percentages (i.e., low concentric loads) may be more work efficient compared to accentuated eccentric loading with higher concentric loads (upon 75% of the 1-RM). Thus, one potential application of EO-RT may be to retain maximum strength while emphasizing higher movement velocities or reducing volume load according to individual aims or training periodization (Ojasto & Hakkinen, 2009; Wagle et al., 2017).

In addition, both SUB and SUPRA groups showed similar gains in MVIC (19% and 23%, respectively). Which supports previous results in which other authors observed similar gains (18%–23%) in isometric strength after EO-RT with submaximal and supramaximal loading (Hortobagyi et al., 2001; Walker et al., 2016). These changes on MVIC may be due to a decreased neural inhibition and subsequent increases in motor unit discharge rate (Aagaard, 2003). Resulting in higher levels of voluntary muscle activation after accentuated eccentric loading compared to traditional training (Walker et al., 2016). Thereby, the force production enhancement observed may be induced by calcium sensitivity and neural drive increase provided by the EO stimulus (Wagle et al., 2017). This response is similar under both supramaximal and submaximal loading conditions (Duchateau & Baudry, 2014), suggesting that the nervous system employs an unique activation pattern during the eccentric contraction independently of the magnitude used during lengthening (Wagle et al., 2017). Therefore, submaximal loading may be an easier approach, which generates less post-exercise damage and lower injury risk, while providing similar benefits, as showed by our results.

It also should be noted that accentuated eccentric loading elicited an improvement in fatigue resistance (Coratella & Schena, 2016). This was evidenced by Walker et al. (2016, 2020) who found significant improvements in the unilateral knee extension repetition-to-failure test (∼28%) after supramaximal EO-RT with high concentric loads. Our data showed similar improvements (∼24%) when EO-RT with supramaximal loads was performed. However, the workload used in our study was much lower, without requiring reaching concentric muscle failure. In addition, submaximal loading showed greater improvements in local muscle endurance (∼54%), diluting the initial differences with SUPRA and showing possibly greater effects. These changes may be due to the fact that the eccentric contraction leads to an increased and longer neural drive, independently of the load magnitude (Wagle et al., 2017). Which in turn could improve the mechanical efficiency and consequently local muscle endurance (Vogt & Hoppeler, 2014; Coratella & Schena, 2016). Therefore, the use of low concentric intensities and a submaximal accentuated eccentric loading might be a good strategy for all those people and athletes who seek to improve their local muscular endurance. However, future research is warranted to deepen these findings and to compare these effects with other training methods.

This task-specific neural adaptations may transfer favorably to sporting activities in which the stretch-shortening cycle is involved, and its optimization has a direct impact on performance. Aiming to analyze EO-RT effects on explosive performance, we observed significant improvements in peak concentric power at 40% of the 1-RM in both SUB (10%–16%) and SUPRA (11%–15%) groups. However, significant improvements at medium (60% of the 1-RM) and high intensity (80% of the 1-RM) were only observed in the SUB group (∼10%). Accordingly, Sheppard and Young, (2010), demonstrated a greater concentric performance in the ballistic bench press preceded by an accentuated eccentric action, especially when a submaximal eccentric loading prior to the explosive movement was used (Sheppard et al., 2007). This greater muscle power production observed in the subsequent concentric contraction, may be a mechanism to explain the adaptations on explosive performance. However, this behavior of the stretch-shortening cycle has not been observed when high training loads were used (80/30% 1-RM) in exercises involving an aerial phase (e.g., jump squat) (Moore et al., 2007). Therefore, eccentrically reinforced training to improve explosive performance should consider that wide ROM, high magnitudes of EO, and eccentric loads higher than concentric 1-RM may be inappropriate, probably due to lengthening amortization phase and; subsequently, limiting the use of the stretch-shortening cycle for concentric potentiation (Komi, 1984; Cormie et al., 2008). Therefore, exercises in which EO occurs during a very short action and at high eccentric velocity have been shown to induce adaptations on muscle power and plyometric performance (Wagle et al., 2017; Hernández-Davó et al., 2021; Handford et al., 2022; Maroto-Izquierdo et al., 2022). Thus, similarly to our results, when very short and concentrated EO action occurs at the end of the eccentric phase after a maximum concentric action, as it occurs when flywheel isoinertial technology is used (Martín-Rivera et al., 2022), have been proved to increase muscle power at low (40%–50% 1 -RM), medium (60%–70% 1-RM) and high intensity (80%–90% 1-RM) in athletes (Maroto-Izquierdo et al., 2017a) and physically active people (Fernandez-Gonzalo et al., 2014; Maroto-Izquierdo et al., 2019; 2022). This may be due, moreover, to the high eccentric speeds used and the absence of an aerial phase, which implies that the eccentric-concentric transition was very fast, resulting in a similar time-under-tension compared to flywheel training. Therefore, accentuated eccentric training may be an optimal strategy to increase the stretch-shortening cycle performance.

These training-induced effects on explosive performance are also noticeable in vertical jump height ability. After 10 weeks of accentuated eccentric training, SJ and DJ vertical jump height improved significantly in both SUB (7%–13%) and SUPRA groups (10%–13%). Even so, CMJ improved only after supramaximal loading, but no significant differences were observed in comparing with SUB. Although the magnitude of the EO seems to have a direct impact on the explosive performance (Maroto-Izquierdo et al., 2019), similar changes in vertical jump height have been recently proved with different eccentric loads and different methods to produce overload (e.g., weight releasers, elastic bands, flywheels and electric-motors) (Aboodarda et al., 2013; Maroto-Izquierdo et al., 2017b; Douglas et al., 2018; Maroto-Izquierdo et al., 2022). Consequently, the ability to store and reutilize elastic energy with a shorter amortization phase was attributed to a short eccentric-concentric transition time and high eccentric velocities during EO training (Douglas et al., 2018; Handford et al., 2022).

All these positive changes experienced by functional variables could be linked to increases in muscle mass. Since the potential hypertrophic benefits of eccentric training raise the possibility that skeletal muscle growth may be enhanced by EO-RT (Schoenfeld & Grgic, 2018). Therefore, muscle hypertrophy could be a possible contributor to favorable changes experienced in performance (Wagle et al., 2017). Thus, changes in thigh lean mass were analyzed before and after training intervention. After training, participants in both groups improved significantly the TTLM (SUB: 2.5%; SUPRA: 4.2%), and also at distal (SUB: 4.6%; SUPRA: 3.4%), medial (SUB: 4.1%; SUPRA: 6.2%), and proximal ROIs (SUB: 4.3%; SUPRA: 3.3%). Although TTLM results showed a slight tendency to greater hypertrophic effects after supramaximal loading (most likely small effect), differences between groups at basal level were maintained after training, so there were no differences between submaximal and supramaximal eccentric loading interventions. This trend has also been observed recently (Maroto-Izquierdo et al., 2019), where larger EO percentages (∼50%) induced by an iso-inertial electric-motor device led to TTLM increases of 4.5%, compared to gains of 3.4% induced by lower EO percentages (∼20%). Similarly, high EO of ∼85–45% using isoinertial free-weights has been shown to be the unique strategy inducing changes in lean mass compared to lower percentages of EO (English et al., 2014). We may not found significant differences inasmuch as both SUB and SUPRA used high EO percentages (60% and 90% of the 1-RM, respectively). Therefore, it is expected that EO magnitude has a greater incidence than the loading condition (Maroto-Izquierdo et al., 2019), since positive effects on lean muscle mass have not been found with low EO percentages (i.e., higher concentric loads) (Fisher et al., 2016). However, similar significant increases in muscle mass after accentuated eccentric training have also been shown in other studies in which submaximal or supramaximal loading were used (Friedmann et al., 2004; Friedmann-Bette et al., 2010; Walker et al., 2016). Hence, it seems to indicate that the submaximal or supramaximal eccentric loads during EO-RT does not have a determining effect on the post-training structural adaptations. Therefore, this study does not allow us to know which strategy is best to develop fat-free mass, nor to identify which is the best load on average to increase muscle mass. Firstly, because the ability to produce eccentric force is individual, and therefore, the percentages of EO are different for each participant. And secondly, because to measure the real EO it is necessary to prescribe the training load relative to the maximum absolute intensity for each contraction. Future studies in which eccentric load is prescribed relative to each person’s eccentric ability are warren to analyze the real effect of EO-RT on hypertrophy.

Moreover, previous studies did not found significant differences between traditional training and EO-RT regarding muscle mass, despite the fact that there were significant differences in muscular strength (Brandenburg & Docherty, 2002; Walker et al., 2016) and vertical jump performance (Friedmann-Bette et al., 2010) improvements. These differences might be due to a lack of region-specific consideration in analysis of CSA [20–22]. Previous studies concluded that eccentric-only training led to favor increases in fascicle length and hypertrophy of the distal muscle area, while concentric-only training results in pennation angle increases and greater hypertrophy at the medial level of the muscle (Franchi et al., 2014). In addition, Franchi et al. (2014) (Franchi et al., 2017; Franchi et al., 2018) concluded that EO training-induced effects may be due to in parallel- or in series-specific hypertrophy, resulting in greater increases in fascicle length (Franchi et al., 2018; Benford et al., 2021) and enhanced mechanotransduction activation (Franchi et al., 2018). However, although DXA-derived lean mass measurements are strongly correlated with magnetic resonance imaging-derived measures of muscle volume (Tavoian et al., 2019), DXA presents several limitations, including its inability to separate muscle groups or provide isolated muscle volume analysis, being only able to quantify the lean tissue mass of a given transverse section of the body. Moreover, error in repeated measurements is not only attributed to machine and evaluator error but also to exercise training or dietary interventions (Tavoian et al., 2019). These changes can impact X-ray attenuation by influencing the relative composition profile of lean tissue mass (especially with respect to fluid content), thus limiting DXA accuracy to quantify muscle-specific gains (Tavoian et al., 2019). Nonetheless, in the present study, 1 week was allowed between the last training session and the post-training DXA scan to re-establish muscle water content levels and allow residual blood to be removed. In any case, future studies should consider using other assessment methodologies such as ultrasound to analyze the architectural changes induced by both EO training strategies.

TTLM increases might be explained by growth factor- and myokine-induced muscle remodeling, activation of myoblast proliferation, or muscle proteome modifications after EO exercise, which may systematically enhance the anabolic environment (Hody et al., 2019). The increased mechanical tension during lengthening during EO training as well as the stimulation of the concentric contraction could induce enhancements in factors involved in anabolic signaling (Yarrow et al., 2007; Ojasto & Hakkinen, 2009; Friedmann-Bette et al., 2010; Walker et al., 2017). Friedmann-Bette and co-workers (2010) found changes in androgen receptor content only after EO-RT, which may influence the effects of serum hormones like testosterone in stimulating muscle protein synthesis. Additionally, insulin-like growth factors such as IGF-1 and myogenic regulatory factors, suggest an increase in satellite cells activation and proliferation, which were also observed only after EO-RT (Friedmann-Bette et al., 2010). These changes in anabolic signaling have been shown to induce morphological changes after EO training (Wagle et al., 2017). Chiefly, within faster muscle fiber types, increasing Type IIx and IIa specific CSA (Friedmann-Bette et al., 2010), and secondly, reducing Type I fiber-type percentage and enhancing Type IIx and IIa fiber-type percentage in muscle groups involved in EO-RT (Friedmann et al., 2004). However, as far as our knowledge is concerned, it was unknown whether there are differences in anabolic signaling between submaximal and supramaximal loading during accentuated eccentric training. The results of this study showed significant increases in IL-6 and IGF-1 after training in both SUB and SUPRA groups, although no differences between groups were detected. These anabolic signaling increases are related to subsequently muscle protein synthesis stimulation, and may be the reason for the greater likely muscle hypertrophic effect of EO-RT (Wagle et al., 2017). Even though, no significant correlations were found between increases in muscle mass and changes in anabolic signaling for any group.

Despite the fact that the results of this study indicated that there were no training-induced differences among groups, and therefore, EO-RT with submaximal loads is effective in promoting neuromuscular and structural changes with less total mechanical work, more information is needed to clarify these results. Indeed, caution should be exercised when interpreting the results of this study, as a limitation is that we are unaware of whether a larger eccentric load (e.g., 140% of concentric 1RM) would have induced greater changes than those observed in the SUPRA group. Moreover, although results obtained through DXA analysis are correlated with Magnetic Resonance Imaging and represents a valid approach to estimate muscle mass, we did not include any other architectural parameter among our variables such as fascicle length. Furthermore, although training-induced adaptations were similar between groups, the measurement of muscle activation during exercise could provide deeper insights into the comparison between different loading strategies. However, muscle activation during exercise has not been measured in this work. Therefore, one of the limitations of this study is the lack of inclusion of other physiological parameters to provide more information on the effects observed, as well as the performance of muscle biopsies to analyze training-induced effects on muscle fibers and their distribution. Finally, a larger sample size that allows to compare the adaptations with other training groups without EO (e.g., 30/30% of the 1-RM) and with different loading approaches. Future studies should focus on the analysis of the EO-RT optimal load that makes possible to achieve the greatest adaptations with the lower total mechanical work, and also the underpinning mechanical and physiological mechanisms that allow the potentiation of the subsequent concentric phase.

In conclusion, 10 weeks of EO-RT in physically active, healthy young men induced significant effects in maximum dynamic strength and maximal voluntary isometric contraction force production, local muscle endurance, muscle power at different loads, vertical jump performance and lean mass in both submaximal (90/30% 1-RM) and supramaximal (120/30% 1-RM) loading conditions. In addition, similar serum hormonal responses were observed between groups, inducing increases in IL-6 and IGF-1. The results of this study showed that accentuated eccentric training with submaximal loads is effective in promoting neuromuscular and structural adaptations, involving greater safety and less total mechanical work. However, supramaximal loading strategy seems to be more favorable for increasing the 1-RM.

## Data Availability

The raw data supporting the conclusion of this article will be made available by the authors, without undue reservation.
