# Supplementary material for: Effects of submaximal and supramaximal accentuated eccentric loading on mass and function
Source: Front Physiol. 2023 Jun 28;14:1176835. doi: 10.3389/fphys.2023.1176835 (PMC10337133; doi:10.3389/fphys.2023.1176835)
Supplement: Supplementary file 1 [file Table1.DOCX]

**Supplementary Table 1.** Perceived muscle soreness (VAS 0-10) mean ± SD values for both submaximal (SUB) and supramaximal groups (SUPRA) immediately after training (0h) and after 2-, 24- and 48-h post-training, *p* value for the comparison between SUB and SUPRA values, effect size (ES) and mean differences and 95% CI are shown for each session and moment.

|  | **SUB** | **SUPRA** | ***p*** | **ES** | **Mean (95% CI)** |
| --- | --- | --- | --- | --- | --- |
| **Session 1** |  |  |  |  |  |
| VAS(0-10) 0 h | 2.8 ± 2.4 | 3.5 ± 2.1 | 0.467 | 0.31 | 0.7 (-1.3 - 2.6) |
| VAS(0-10) 2 h | 2.5 ± 1.9 | 3.0 ± 1.3 | 0.560 | 0.25 | 0.4 (-1.0 - 1.9) |
| VAS(0-10) 24 h | 2.2 ± 1.9 | 2.5 ± 2.6 | 0.703 | 0.16 | 0.4 (-1.5 - 2.3) |
| VAS(0-10) 48 h | 1.2 ± 1.6 | 1.2 ± 1.4 | 0.963 | 0.02 | 0.0 (-1.3 - 1.3) |
| **Session 10** |  |  |  |  |  |
| VAS(0-10) 0 h | 2.5 ± 2.2 | 3.2 ± 1.8 | 0.453 | 0.32 | 0.7 (-1.1 - 2.5) |
| VAS(0-10) 2 h | 1.7 ± 1.4 | 2.5 ± 1.8 | 0.216 | 0.54 | 0.8 (-1.1 - 2-5) |
| VAS(0-10) 24 h | 1.2 ± 1.7 | 2.0 ± 1.5 | 0.244 | 0.50 | 0.8 (-0.6 - 2.2) |
| VAS(0-10) 48 h | 0.8 ± 1.7 | 0.7 ± 0.9 | 0.841 | 0.08 | 0.1 (-1.1 - 1.3) |
| **Session 20** |  |  |  |  |  |
| VAS(0-10) 0 h | 1.8 ± 2.0 | 2.6 ± 2.0 | 0.907 | 0.39 | 0.8 (-1.1 - 2.7) |
| VAS(0-10) 2 h | 0.9 ± 1.4 | 1.9 ± 2.1 | 0.234 | 0.56 | 0.9 (-0.6 - 2.6) |
| VAS(0-10) 24 h | 0.4 ± 0.6 | 0.8 ± 0.8 | 0.257 | 0.54 | 0.4 (-0.3 - 1.1) |
| VAS(0-10) 48 h | 0.2 ± 0.2 | 0.4 ± 0.6 | 0.337 | 0.46 | 0.2 (-0.2 - 0.6) |
